# Case Report: Thromboembolism and Hemorrhagic Pericardial Effusion—The Janus Face of Primary Pericardial Angiosarcoma

**DOI:** 10.3389/fcvm.2020.618146

**Published:** 2021-01-15

**Authors:** Fei F. Chen, Shu F. Jiang, Chang Dong, Ying Che, Lin Y. Du, Zhi Y. Li, Zhi Q. Yang, Yi C. Zhao, Ying Liu

**Affiliations:** ^1^Department of Cardiology, The First Affiliated Hospital of Dalian Medical University, Dalian, China; ^2^Department of Respiratory, The First Affiliated Hospital of Dalian Medical University, Dalian, China; ^3^Department of Ultrasonography, The First Affiliated Hospital of Dalian Medical University, Dalian, China; ^4^Department of Radiology, The First Affiliated Hospital of Dalian Medical University, Dalian, China

**Keywords:** cardiac angiosarcoma, pulmonary thromboembolism, pulmonary metastasis, hemoptysis, cardiac tamponade

## Abstract

**Background:** Primary cardiac angiosarcomas, especially those originating in the pericardium, are extremely rare and aggressive tumors with poor prognosis. These types of malignant tumors have diverse clinical presentations and are often masked by other comorbidities.

**Case Summary:** Our hospital reported a 59-year-old woman who initially presented with pulmonary thromboembolism (PTE) and was subsequently treated with low-molecular-weight heparin. However, she experienced acute pericardial tamponade after anticoagulation therapy, where no obvious mass was primarily identified upon imaging, both in the pericardium or within the heart. Emergency pericardiocentesis and drainage were performed, where a total of 210 mL of bloody effusion was drained. Four months later, she was hospitalized with progressive hemoptysis and dyspnea. A large mixed mass occupying the right pericardium was later identified by coronary computed tomography angiography (CCTA). The mass was consistent with the right atrium, with heterogeneous thickened pericardium and localized moderate pericardial effusion. CCTA and positron emission tomography scans later showed metastases in both lungs and bilateral pleura. Nodules in hilar and mediastinal lymph nodes were also significant. Ultrasound-guided biopsy was performed, and the patient was ultimately diagnosed with an angiosarcoma based on final positive results for both CD31 and CD34 markers. The patient refused chemotherapy and passed away while waiting for her pathology results. The patient survived for 6 months since the first reported episode of PTE.

**Conclusions:** Our case indicates that patients presenting with both embolism and hemorrhage should urgently be channeled to a clinical specialist to confirm any malignant etiology. This would be beneficial to confirm an early diagnosis and lengthen the duration of patient survival. However, the diagnosis of primary cardiac angiosarcoma is still challenging and requires multiple imaging modalities and biopsies in order to assist the accurate diagnosis of disease and achieve effective patient management.

## Introduction

Angiosarcomas are the most common histological subtype of sarcomas and usually occur in middle-aged patients, with a ratio of 2:1 between men and women. The right atrium (RA) is the usual location of the involvement of cardiac angiosarcomas (1). The primary features of angiosarcoma include rapid proliferation, extensive infiltration, and distant metastasis. Metastatic disease is very frequent at the time of diagnosis (seen in 66–89% of patients), mainly locally (mediastinal lymph nodes, lungs, and vertebra) (2). However, the diagnosis still remains a challenge because of its long-time silent evolution and variability of clinical presentation. The median survival time between the onset of symptomatic disease and diagnosis in the present series was 5 months, with a range of 2–12 months in various patients of similar disease etiology (1). Furthermore, immunohistochemical staining studies show positive for CD31, CD34, and factor VIII, vascular endothelial cell markers.

## Case Presentation

**Figure 1:**
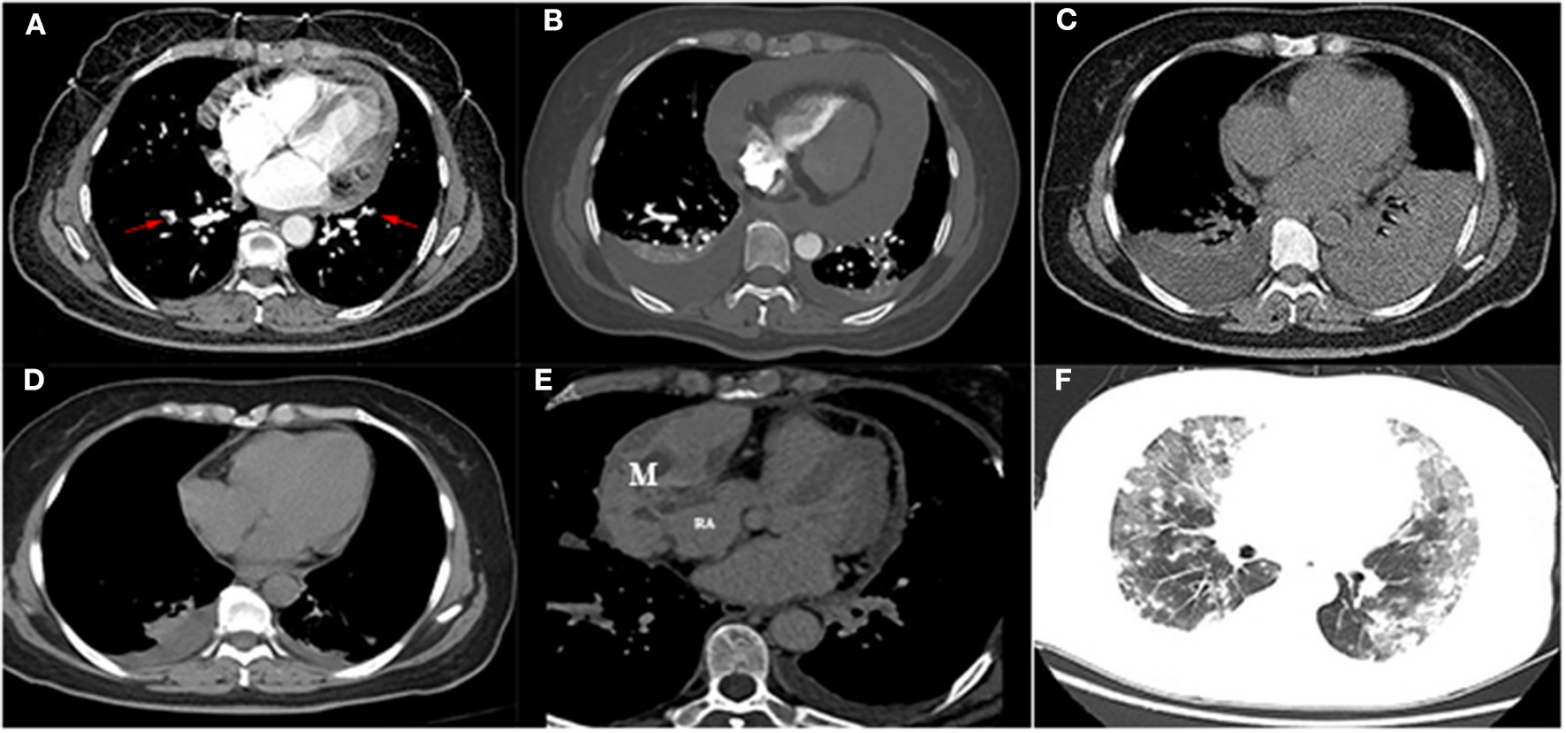
**(A)** Computed tomography pulmonary angiography showed multiple embolisms of bilateral pulmonary arteries (arrow) with a mild-sized pericardial effusion. **(B)** Computed tomography showed a severe-sized pericardial effusion and bilateral pleural effusion after heparin anticoagulation. **(C)** The left pleural effusion was increased after pericardial drainage. **(D)** Computed tomography revealed mild bilateral pleural effusion and pericardial effusion after drainage. **(E)** Contrast-enhanced computer tomography showed a mixed mass located in the right pericardium (4.5 × 8.5 cm), and the boundary was unclear between the mass and the right atrial wall, heterogeneous thickened pericardium, and moderate pericardial effusion locally. **(F)** Multiple metastatic nodules surrounded by a wide range of ground-glass-like effusion (diffuse alveolar hemorrhage) were identified in the bilateral lungs. M, mass; RA, right atrium.

A 59-year-old woman with no past medical history presented with 1-week symptoms of atypical chest pain and mild dyspnea. Upon physical examination, her heart rate, respiration rate, blood pressure, and body temperature were 102 beats per minute (bpm), 22 breaths per minute, 110/60 mm Hg, and 36.1°C, respectively. No moist rales or wheezing sound in lungs and no cardiac murmur or pericardial rub were detected. Laboratory tests showed the level of d-dimer as 6,440 μg/L (reference range, 0–550 μg/L) and brain natriuretic peptide as 107.52 ng/L (reference range, 0–100 ng/L). Arterial blood gas analysis in room air showed severe hypoxemia (pH 7.393, Pao_2_ = 60 mm Hg, Paco_2_ = 43.3 mm Hg, ${\text{HCO}}_{3}^{-}$ = 25.8 mmol/L). Myocardial enzymes, tumor markers, thyroid function tests, T-SPOT, tuberculosis antibody, hepatitis virus, human immunodeficiency virus, syphilis, and autoimmune panel were all negative. An electrocardiogram showed a fast sinus rhythm of 102 bpm, with flat T-wave in precordial leads. Computed tomography pulmonary angiography (CTPA) showed multiple embolisms of bilateral pulmonary arteries and a mild-sized pericardial effusion (Figure 1A). Color Doppler ultrasound showed left calf muscular venous thrombosis. Upon clinical examination and tests, the patient was diagnosed of acute pulmonary thromboembolism (PTE) (intermediate risk) and left muscular calf vein thrombosis (diagnosed on December 16, 2019). First line of treatment included hypodermic injection of low-molecular-weight heparin as anticoagulation therapy.

However, 2 days posttherapy, the patient complained of progressive development of dyspnea, fatigue, chest tightness, and palpitations, to later experience transient loss of consciousness. Her heart rate increased to 125 bpm, whereas blood pressure and pulse pressure were slightly decreased (96/69 and 27 mm Hg, respectively). Jugular venous distention and muffled heart sounds were present. Both transthoracic echocardiogram (TTE) and thoracic CT were significant for severe large-scale pericardial effusion (Figure 1B). No obvious mass was identified either in the pericardium or heart. Therefore, acute pericardial tamponade was considered as a differential diagnosis. Emergency pericardiocentesis and drainage were performed, draining 210 mL of bloody effusion in total. Repeated thoracic CT the following day was significant for bilateral pleural effusion, which was greatly increased in amount (Figure 1C). Further left thoracentesis and thoracic drainage were performed, draining another 710 mL of pleural effusion (the first two times, collected effusion was bloody; the third time, effusion was examined as pale yellow). Symptoms of dyspnea and palpitations were also immediately improved after a third round of drainage. Additional pericardial effusion cultures for bacteria and acid-fast bacilli were repeated twice, obtaining negative reports each time. Repeated cytological examination of the pericardial effusion did not show any malignant cells. Repeated thoracic CT prior to discharge showed mild bilateral pleural effusion and pericardial effusion (documented on December 30, 2019; Figure 1D). Patient was followed up in the outpatient department.

Half a month later (January 15, 2020), although she was asymptomatic, her levels of d-dimer were spiked to 10,740 μg/L. TTE showed a mild pericardial effusion. Given that PTE is prone to relapse, she suffered an acute pericardial hemorrhage after anticoagulation, where low-dose rivaroxaban (10 mg every day) was later administered from January 15, 2020, onward. The levels of d-dimer were decreased significantly (1,450 μg/L) after 2 months of oral rivaroxaban, and there were no bleeding events recorded during this period.

**Figure 2:**
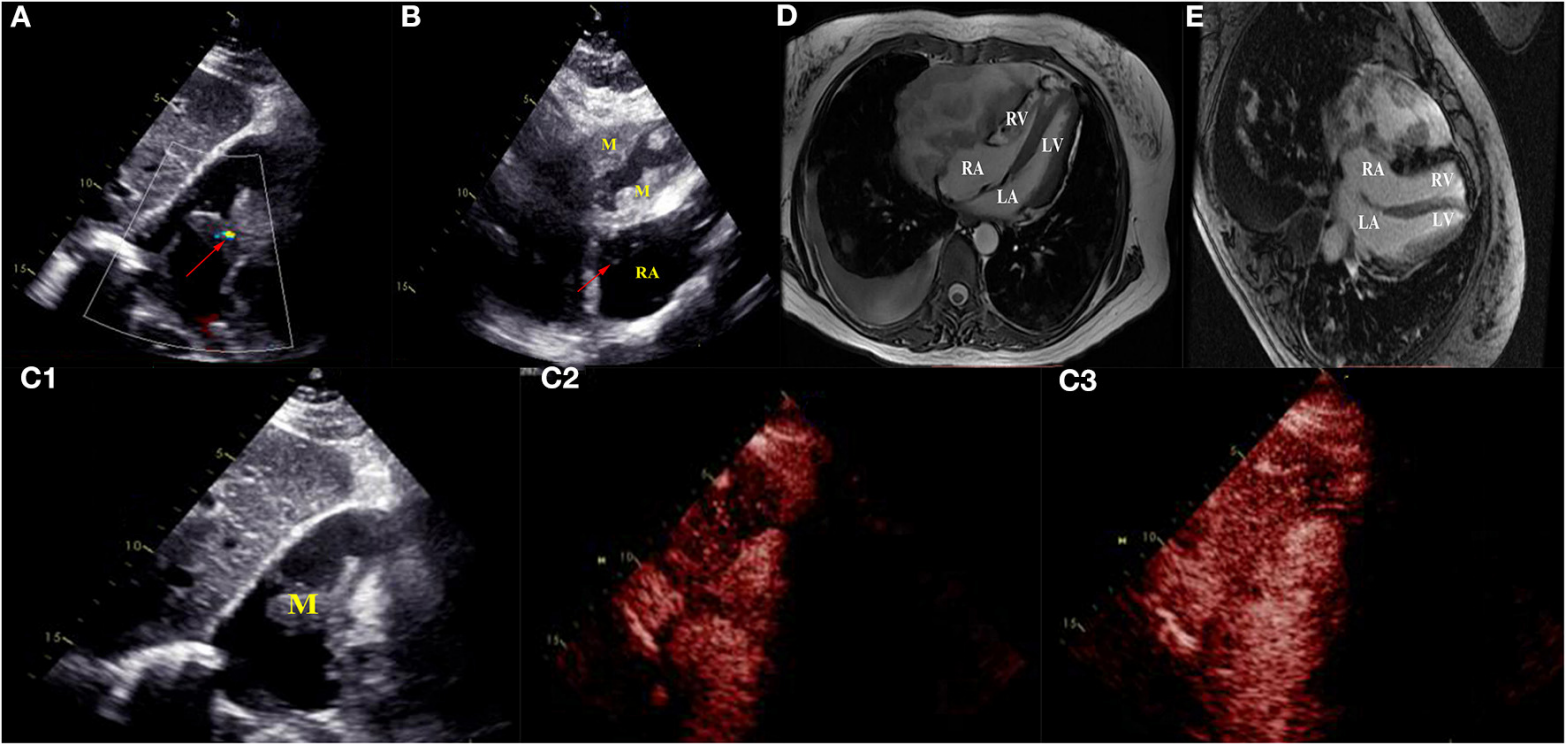
Transthoracic echocardiogram revealed a huge mass in the pericardium surrounding and compressing the right atrium and right ventricle **(A,B)**. Mild blood flow signals (arrow) were seen in the mass **(A)** (May 20, 2020). The tumor was significantly larger than before and strip-like hyperechoism (arrow) inside the right atrium **(B)** (June 18, 2020). Contrast-enhanced ultrasound revealed the mass transferred right atrium, and the mass was rapidly filled with contrast agent **(C1–C3)** (May 20, 2020). Axial Fiesta **(D)** and 4CH-2D **(E)** images of cardiac magnetic resonance showed an irregular, large tumor (size up to 10 × 5.5 cm) with heterogeneous distinction diffusely within the pericardium (10 × 5.5 cm), mainly locating outside of right cardiac system, and compressed the right heart. RA, right atrium; RV, right ventricle; LA, left atrium; LV, left ventricle; M, mass; RA, right atrium.

**Figure 3:**
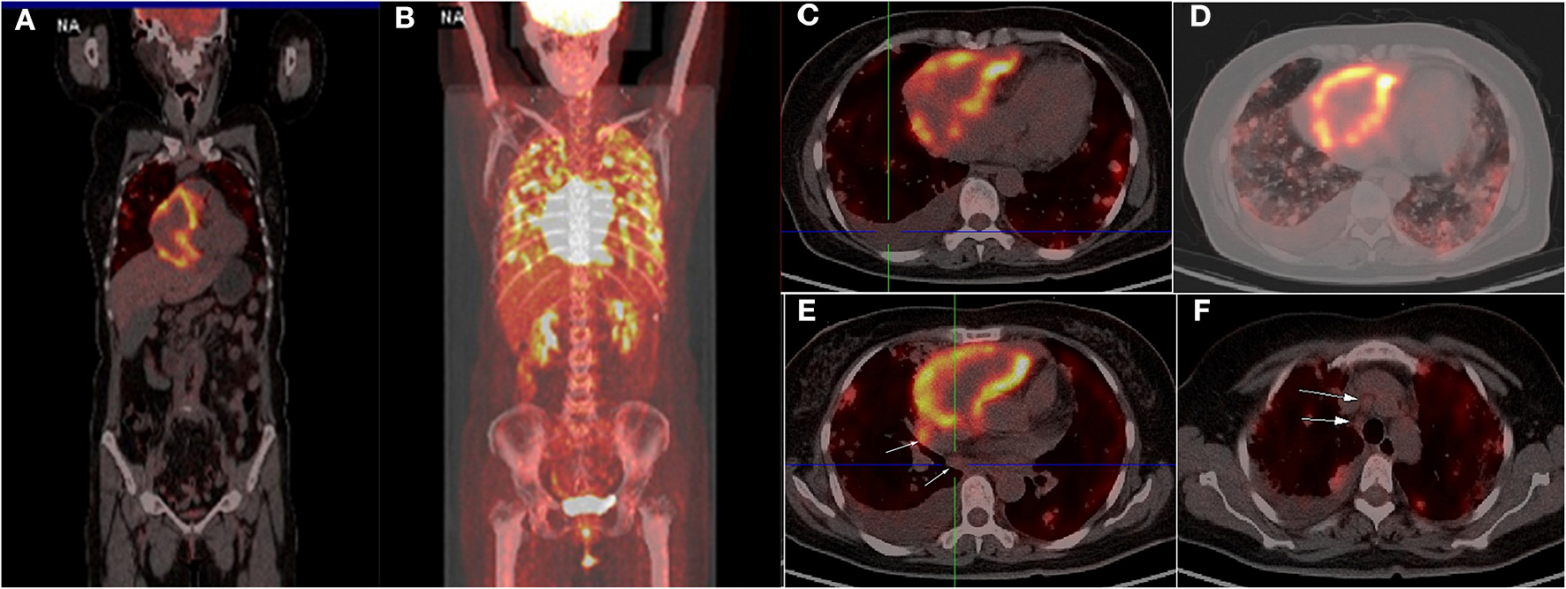
Positron emission tomography scan showed a heterogeneous mass at the right margin of pericardium with circular hypermetabolic uptake of fluorodeoxyglucose-18, poorly demarcated from the right atrium **(A–C)**. Distant metastases were found in both lungs, bilateral pleural **(A,D)**, nodules in hilar and mediastinal lymph **(E,F)** (arrow).

**Figure 4:**
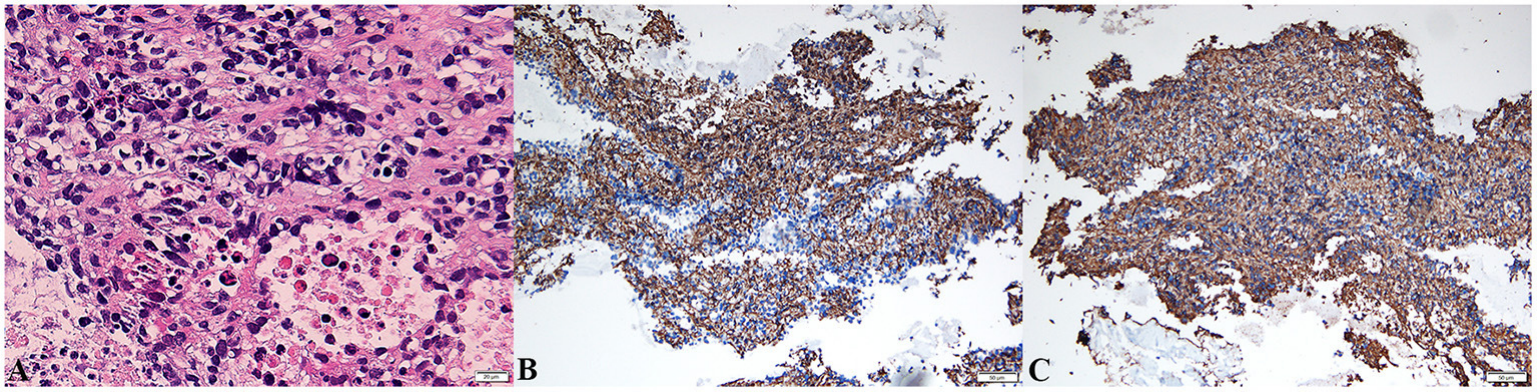
Biopsy of pericardial mass showed pleomorphic spindle and ovoid tumor cells with atypia and mitotic [hematoxylin–eosin stain, original magnification ×400, **(A)**]. The malignant cells were strongly positive for CD31 [blue, ×200, **(B)**] and CD34 [blue, ×200, **(C)**] in immunohistochemistry.

Four months later, the patient complained of progressive hemoptysis and dyspnea (May 2, 2020). She did not stop taking the administered oral rivaroxaban until the embolisms were absent by CTPA on May 18, 2020. However, CTPA also showed a large mixed mass, occupying the right pericardium (4.5 × 8.5 cm; Figure 1E), and she was readmitted. On physical examination, mucocutaneous color was pale, and medium moist rales in lungs was detected. The levels of CA-125 and neuron-specific enolase (NSE) were markedly elevated (117.5 U/mL [reference range, 0–35 U/mL]; 46.96 ng/mL [reference range, 0–24 ng/mL], respectively). The hemoglobin content was 68 g/L (reference range, 115–150 g/L); the plasma d-dimer level was 4,160 μg/L; arterial pressure of oxygen was 56 mm Hg (reference range, 80–100 mm Hg). Dual-source coronary CT angiography (CCTA) showed the mass infiltrated and communicated with the RA, with heterogeneous thickened pericardium and localized moderate pericardial effusion (Supplementary Movie 1). No stenosis was seen in the coronaries, and there was no communication of the tumor with the coronaries. CCTA also showed a mild left pleural effusion, enlarged mediastinal and hilar lymph nodes, multiple and scattered ground-glass opacity [diffuse alveolar hemorrhage (DAH)] nodules over bilateral lung field, and multiple bipleural nodules (Figure 1F, May 21, 2020). TTE showed a huge mass in the pericardium, surrounding and compressing the RA and right ventricle. Mild blood flow signals were also observed in the mass (Figure 2A) (May 20, 2020). The tumor was significantly larger than before with a strip-like hyperechoism within the RA (Figure 2B, recorded on June 18, 2020). Contrast-enhanced ultrasound showed the mass and chamber were filled with contrast agent almost simultaneously (Figures 2C1–C3 and Supplementary Movie 2). Axial Fiesta (Figure 2D) and 4CH-2D (Figure 2E) of cardiac magnetic resonance (CMR) showed an irregular, large tumor (size up to 10 × 5.5 cm) with heterogeneous distinction diffusely within the pericardium (10 × 5.5 cm), mainly located outside the right cardiac chamber system, compressing the right heart. Positron emission tomography scan exhibited a heterogeneous mass located at the right margin of pericardium, poorly demarcated from the RA and superior vena cava (Figures 3A–C). Distant metastases were found in both lungs and bilateral pleura (Figures 3B,D), nodules in hilar and mediastinal lymph nodes (Figures 3E,F).

**Figure 5:**
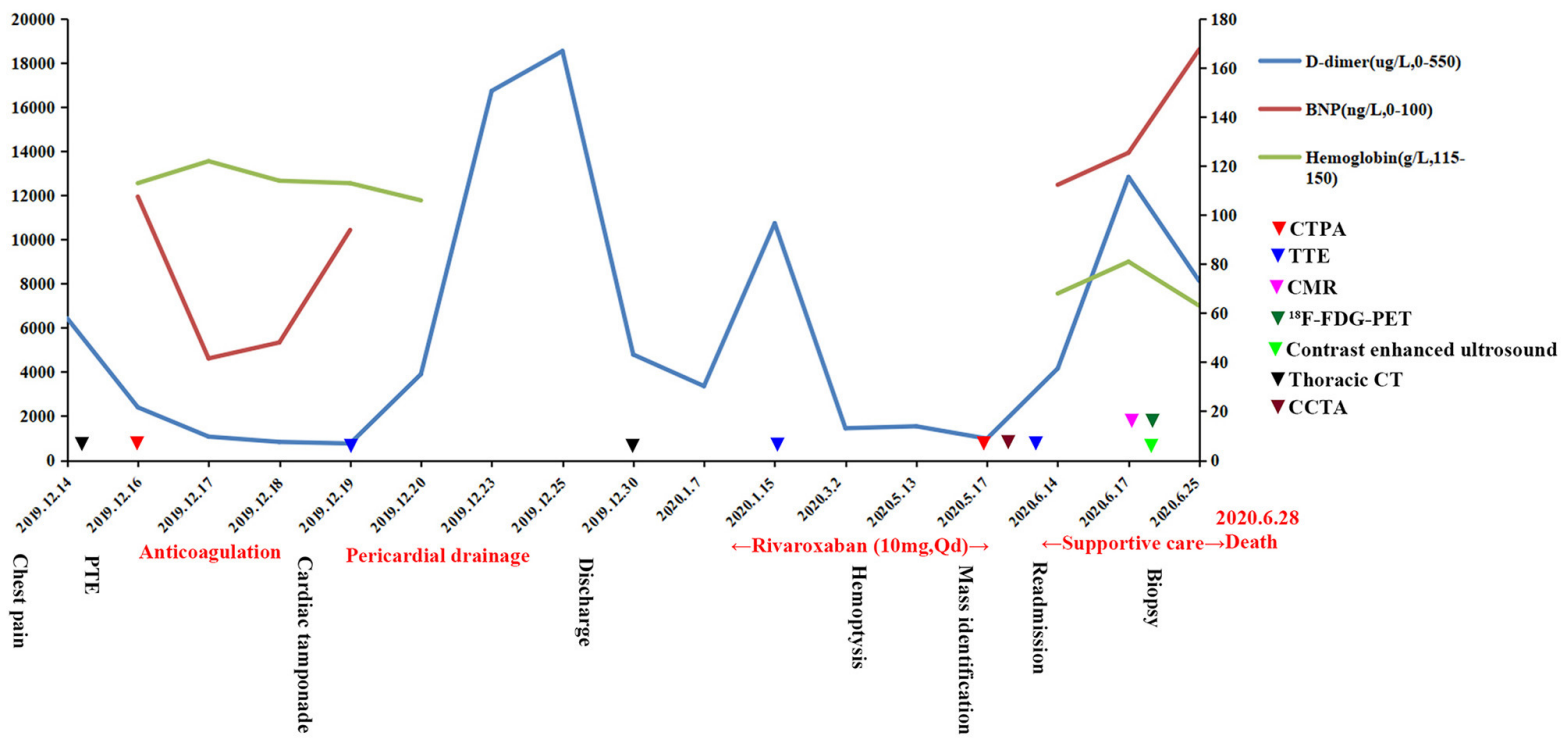
Progression and examination of the clinical course. PTE, pulmonary thromboembolism; BNP, brain natriuretic peptide; CTPA, computed tomography pulmonary angiography; TTE, transthoracic echocardiogram; CMR, cardiac magnetic resonance; PET, positron emission tomography; CCTA, coronary computed tomography angiography.

Ultrasound-guided biopsy was performed for a definitive diagnosis. The biopsy specimen showed a malignant tumor in histopathologic examination (Figure 4A) and positive immunohistochemical staining for CD31 (Figure 4B) and CD34 (Figure 4C), but negative for CEA, CK, CK5/6, CR, D2-40, desmin, and MC, indicating poorly differentiated angiosarcoma. Surgery was not a suitable option for the patient because of metastasis. Unfortunately, she refused treatment with radiotherapy, chemotherapy, or immunotherapy, where the clinical condition of the patient deteriorated rapidly after. She died on June 28, 2020, while waiting for the pathologic results due on June 29, 2020. The patient survived for 6 months from the first episode of PTE. The progression and examination of the clinical course are shown in Figure 5.

## Discussion

Cardiac angiosarcoma is most commonly found in RA and rarely occurs in the epicardium, pericardium, or left ventricle (3). (The common tumors of pericardium are shown in Supplementary Table 1). The clinical manifestations of pericardial angiosarcoma include (i) repeated medium to large amounts of hemorrhagic pericardial effusion; (ii) one or more irregular masses extending along the pericardium, surrounding or compressing the heart (4); (iii) and thickened pericardium, which can appear as a linear contrast enhancement on CMR with radiation from the epicardium to the pericardium (5). Right heart failure and cardiac tamponade are also common complications (6). In this patient, an acute cardiac tamponade occurred during anticoagulation therapy. The mass was diffused along the pericardium, combined with the thickened pericardium and pericardial effusion. Also, the mass compressed the right cardiac system, communicating with the RA. Thus, it was considered that the mass originated from the pericardium.

Morphologically, angiosarcoma is a hemorrhagic mass with ill-defined margins and multiple necroses, calcification, and lobulation. The most common complaint is hemoptysis when the patient has developed pulmonary metastasis. Most angiosarcomas are thought to originate from the vascular endothelium and spread through the bloodstream to the lung, eventually eroding and obstructing the pulmonary vessels. Therefore, either DAH or PTE is often one of the first and foremost presenting manifestations (3). DAH is an uncommon condition in which blood floods the alveoli. The causes of DAH include Wegener granulomatosis, microscopic polyangiitis, Goodpasture syndrome, connective tissue disorders, antiphospholipid antibody syndrome, infectious or toxic disorders, and neoplastic conditions (7). Pulmonary metastasis of angiosarcoma is a rare cause of DAH. The most common known primary site is the heart (8). Radiologically, it most often comprises the presence of two heterogeneous lesions: bilateral, peripheral nodules and ground-glass shadow. These lesions are rapidly aggravated in the course of 1–2 weeks. In our patient, she presented progressive dyspnea, hemoptysis, anemia, and refractory hypoxemia. The clinical outcome was poor, although she underwent aggressive treatment including drug hemostasis, blood transfusion, and high-flow oxygen inhalation. In addition, to our knowledge, it was the first time to report both the presence of DAH and PTE in pulmonary metastases of angiosarcoma.

Looking back on the whole course of the disease, several signs suggested that it might be a tumor. First, the patient initially presented with PTE, and it is generally recognized that cancer has a high risk of PTE. Li et al. reported that pulmonary embolism (PE) is a frequent finding, with an overall incidence of 3.7% (1,172 of 31,294), ranging from 0–23.7% in lung cancer patients (9). PE resulted from a combination of the following: vascular endothelial damage, stasis of blood flow, and hypercoagulability. These factors appear to be present in cancer patients. Second, the patient had acute pericardial hemorrhage after anticoagulation, which accurately fits the characteristic of a typical tumor. Because most angiosarcomas show high levels of blood perfusion, they are prone to hemorrhage. Cornily et al. revealed malignant diseases are the leading etiology of tamponade in their population (74/114 patients, 65%) exceeding other causes by far (10). Cardiac tamponade is a serious event during cancer development, and its prognosis seems to be independent to the development of a primitive tumor (10). Third, a small pericardial effusion (not mass) was still detected by TTE in the 2nd week after discharge. But it was considered as incomplete absorption of pericardial effusion at that time. A small primary pericardial angiosarcoma is therefore likely to yield a missed diagnosis or misdiagnosis. Therefore, it is important to pay attention to screen for potential tumors in patients presenting with both embolism and hemorrhage.

Primary cardiac angiosarcomas have a poor prognosis with median survival rates ranging from 6 to 11 months (11). Survival after surgical intervention has a median of 14 months (11), while with systemic metastasis has only 3.8 ± 2.5 months' difference without surgical resection (12). Surgery is a commonly chosen therapy, particularly in the setting of localized disease, in that it seems to provide the best option for palliative care and potential cure (13). Although most angiosarcomas are resistant to both chemotherapy and radiation, they are nonetheless important in the treatment of tumors due to the high probability of the patient developing further metastases (14). Cyclophosphamide, cisplatin, dacarbazine, doxorubicin, ifosfamide, mitomycin-C, paclitaxel, and vincristine are commonly prescribed agents in standard chemotherapy (15). Because of our patient refusing gold-standard chemotherapy and radiotherapy, the tumors rapidly effused and aggravated during her last 1–2 weeks of survival. She also lost the chance of receiving surgical intervention because metastases had already developed. Unfortunately, she died while waiting for her pathologic results. The patient survived for 6 months from the first episode of PTE and only 40 days from the first identification of the mass.

In conclusion, patients presenting with both embolism and hemorrhage should prompt the clinician to seek for a malignant etiology. DAH is a rare presentation of pulmonary metastases of angiosarcoma, and the most common known primary site is the heart. However, the diagnosis of primary cardiac angiosarcoma is still challenging, and it needs multiple imaging techniques and biopsies to assist in its effective diagnosis.

## Data Availability Statement

The original contributions generated for this study are included in the article, further inquiries can be directed to the corresponding author.

## Ethics Statement

Written informed consent for the present report was obtained from the patient's husband.

## Author Contributions

YL contributed to the conception of the case report. FC and SJ contributed to the management of the patient and manuscript writing. CD contributed to the pulmonary embolism treatment of the patient and manuscript preparation. YC and LD performed the the ultrasound-guided biopsy. ZL and ZY contributed to the imageological examination. YZ contributed to the clinical data collection. All authors contributed to the article and approved the submitted version.

## Conflict of Interest

The authors declare that the research was conducted in the absence of any commercial or financial relationships that could be construed as a potential conflict of interest.
