# Supplementary material for: Case Report: Thromboembolism and Hemorrhagic Pericardial Effusion—The Janus Face of Primary Pericardial Angiosarcoma
Source: Front Cardiovasc Med. 2021 Jan 15;7:618146. doi: 10.3389/fcvm.2020.618146 (PMC7843435; doi:10.3389/fcvm.2020.618146)
Supplement: Supplementary file 3 [file Table_1.DOCX]

**Supplementary Table 1 The classification of tumors of pericardium**

| **Histologic Type** | **Benign or malignant** | **Age** | **Sex** | **Metastasis** | **Incidence** | **Survival time** |
| --- | --- | --- | --- | --- | --- | --- |
| **Primary pericardial tumors** |  |  |  |  |  |  |
| Germ cell tumors |  |  |  |  |  |  |
| Teratoma^[1]^ | Mostly benign | Fetus, neonate | Slight predominance in males | No | 0.15 %–0.2%  (autopsy incidence) | Good |
| Yolk sac tumor ^[2]^ | Mostly benign | Fetus, children | Slight predominance in females | Yes | Rare | Good |
| Sarcoma |  |  |  |  |  |  |
| Synovial sarcoma^[3]^ | Malignant | Adolescence, early youth | Mostly in males | Yes | 5% | 27 months |
| Angiosarcoma^[4]^ |  | Adults, mostly <65yrs | Male:female 2:1 | Yes | 0.0001-0.030%  (autopsy incidence) | 6-11 months |
| Rhabdomyosarcoma^[5]^ |  | Fetus, children | - | Yes | - | Poor |
| Undifferentiated pleomorphic sarcoma^[2]^ | Malignant | Adults | - | - | Rare | Poor |
| Malignant mesothelioma^[6]^ | Malignant | 3–80 years | Male:female 2:1 |  | <0.0022% | <6 months |
| Solitary fibrous tumor^[7]^ | Mostly benign | Adults | - | No | Rare | - |
| **Cardiac metastases involving the pericardium**^[8, 9]^ 20-30 times than primary cardiac tumors (2%-18%) | | | | | | |
| Carcinoma of the lung |  |  |  |  |  |  |
| Adenocarcinomas |  |  |  |  |  |  |
| Squamous cell carcinomas |  |  |  |  |  |  |
| Undifferentiated carcinomas |  |  |  |  |  |  |
| Carcinoma of the breast |  |  |  |  |  |  |
| Melanoma |  |  |  |  |  |  |
| Carcinoma of the ovary |  |  |  |  |  |  |
| Carcinoma of the stomach |  |  |  |  |  |  |
| Carcinoma of the prostate |  |  |  |  |  |  |
| Hematologic malignancies |  |  |  |  |  |  |
| The tumors of Pericardium can be metastasised by any malignant neoplasm able to spread to distant sites. | | | | | | |

**Reference**

[1] Cavalcante CTMB, Pinto Júnior VC, Pompeu RG, Férrer JVCC, Cavalcante MB, Araujo Júnior E, Peixoto AB, Castello Branco KM. Early treatment of intrapericardial teratoma: a case presentation and systematic literature review. J Matern Fetal Neonatal Med 2019; 32:2262-2268.

[2] Burke A, Turke F. The 2015 WHO Classification of Tumors of the Heart and Pericardium. Journal of Thoracic Oncology 2016; 11:441-452.

[3] Duran-Moreno J, Kampoli K, Kapetanakis EI, Mademli M, Koufopoulos N, Foukas PG, Kostopanagiotou K, Tomos P, Koumarianou A. Pericardial Synovial Sarcoma: Case Report, Literature Review and Pooled Analysis. In Vivo 2019; 33:1531-1538.

[4] Patel SD, Peterson A, Bartczak A, Lee S, Chojnowski S, Gajewski P, Loukas M. Primary cardiac angiosarcoma-a review. Med Sci Monit 2014; 20:103-109.

[5] Mongé MC, Walterhouse DO, Kalapurakal JA, Rigsby CK, Backer CL. Resection of Intrapericardial Rhabdomyosarcoma in a Child. World J Pediatr Congenit Heart Surg 2016; 7:108-111.

[6] Primary pericardial mesothelioma: a rare entity. Godar M, Liu JH, Zhang PG, Xia Y, Yuan QH. Case Rep Oncol Med 2013; 2013:283601-283603.

[7] Czimbalmos C, Csecs I, Polos M, Bartha E, Szucs N, Toth A, Maurovich-Horvat P, Becker D, Sapi Z, Szabolcs Z, Merkely B, Vago H. Uncommon presentation of a rare tumour - incidental finding in an asymptomatic patient: case report and comprehensive review of the literature on intrapericardial solitary fibrous tumours. BMC Cancer 2017; 17:612-615.

[8] Burazor I, Aviel-Ronen S, Imazio M, Markel G, Grossman Y, Yosepovich A, Adler Y. Primary malignancies of the heart and pericardium. Clinical Cardiology 2015; 37:582-588.

[9] Zitzelsberger T , Eigentler T K , Patrick Krumm, Nikolaou K, Garbe C, Gawaz M, Klumpp B. Imaging characteristics of cardiac metastases in patients with malignant melanoma. Cancer Imaging 2017; 17:19-24.
